# Synthetic dosage lethal (SDL) interaction data of Hmt1 arginine methyltransferase

**DOI:** 10.1016/j.dib.2020.105885

**Published:** 2020-06-21

**Authors:** Dimitris Kyriakou, Mamantia Constantinou, Antonis Kirmizis

**Affiliations:** a EFEVRE TECH LTD, Larnaca, Cyprus; b Department of Biological Sciences, University of Cyprus, 1 University Ave, Nicosia, Aglantzia 2109, Cyprus

**Keywords:** Synthetic dosage lethality, Genetic interactions, Arginine methylation, Hmt1, Post-translational modifications, PRMT

## Abstract

The introduction of methyl groups on arginine residues is catalysed by Protein Arginine Methyltransferases (PRMTs). However, the regulatory mechanisms that dictate the levels of protein arginine methylation within cells are still not completely understood. We employed Synthetic Dosage Lethality (SDL) screening in *Saccharomyces cerevisiae*, for the identification of putative regulators of arginine methylation mediated by Hmt1 (HnRNP methyltransferase 1), ortholog of human PRMT1. We developed an SDL array of 4548 yeast strains in which each strain contained a single non-essential gene deletion, in combination with a galactose-inducible construct overexpressing wild-type (WT) Hmt1-HZ tagged protein. We identified 129 consistent SDL interactions for WT Hmt1-HZ which represented genes whose deletion displayed significant growth reduction when combined with WT Hmt1 overexpression. To identify among the SDL interactions those that were dependent on the methyltransferase activity of Hmt1, SDL screens were repeated using an array overexpressing a catalytically inactive Hmt1(G68R)-HZ protein. Furthermore, an additional SDL control screen was performed using an array overexpressing only the protein tag HZ (His_6_—HA-ZZ) to eliminate false-positive SDL interactions. This analysis has led to a dataset of 50 high-confidence SDL interactions of WT Hmt1 which enrich eight Gene Ontology biological process terms. This dataset can be further exploited in biochemical and functional studies to illuminate which of the SDL interactors of Hmt1 correspond to factors implicated in the regulation of Hmt1-mediated arginine methylation and reveal the underlying molecular mechanisms.

**Specifications Table**

**Table:** 

**Subject**	Biological Sciences
**Specific subject area**	Functional genomics, genetics, epigenetics
**Type of data**	Tables and figures
**How data were acquired**	Synthetic Dosage Lethality assay [1,2] was performed by BM3-BC yeast handling robot (S&P Robotics, Inc.). Colony size of each yeast strain was analysed by spImager (S&P robotics Inc). The data were analysed as previously described in [3].
**Data format**	Raw, analysed and filtered
**Parameters for data collection**	Three independent yeast strain arrays were generated by combining 4548 non-essential yeast gene deletion strains with an inducible overexpression of either 1) wild-type Hmt1-HZ, 2) catalytically inactive Hmt1(G68R)-HZ [4], or 3) the HZ protein tag alone. Each array was then cultured in non-inducing (glucose or raffinose media) and inducing conditions (galactose media) and the colony size of each yeast strain was quantified.
**Description of data collection**	Yeast colony size was determined using spiImager (S&P Robotics, Inc.) and saved as excel files (Table S1). Yeast strains displaying a growth reduction greater than 30% in inducing compared to non-inducing conditions were designated as SDL interactions (Tables S2–4). SDL interactions derived by overexpression of WT Hmt1-HZ were then filtered using the SDL interactions generated by the two control screens (Table 1, Fig. 1) to identify high-confidence SDL interactions of Hmt1 which are dependent on its methyltransferase activity (Table 2).
**Data source location**	Institution: Department of Biological Sciences, University of Cyprus
	City/Town/Region: Aglantzia, Nicosia
	Country: Cyprus
	Latitude and longitude (and GPS coordinates) for collected samples/data: 4CW5+87
**Data accessibility**	With the article

**Value of the Data**

• The data reported will be useful for identifying putative novel regulators of protein arginine methylation, substrates of arginine methyltransferases and biological processes that are affected by arginine methylation.
• The generated dataset is helpful for advancing the work of researchers who are investigating protein function and specifically the biological role of protein post-translational modifications.
• These data will direct future studies on the biochemical and molecular mechanism of arginine methylation.
• The strategy for generating the SDL interaction data of Hmt1 can be extended to investigate other post-translational modifications and their associated enzymes.
• These data describe new collections of mutant yeast strains that could be useful to researchers using *S. cerevisiae* as a model system.

## 1 Data

**Fig. 1:**
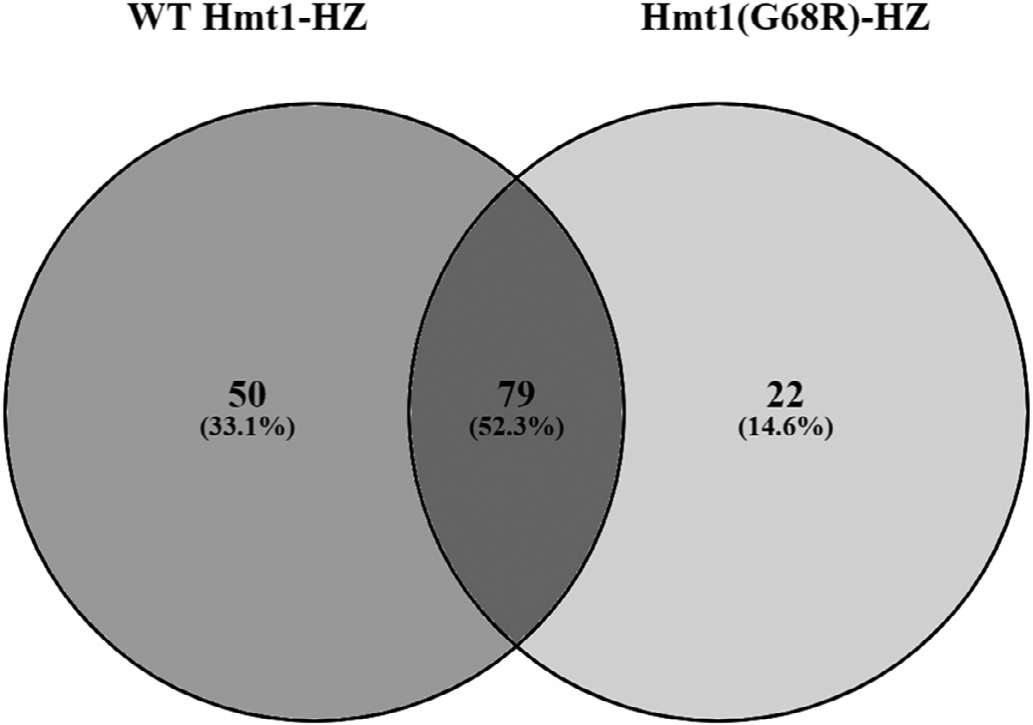
**Comparison of SDL interactions of wild-type Hmt1 and catalytically inactive Hmt1(G68R).** Venn diagram indicating the total SDL interactions generated from the WT Hmt1-HZ and Hmt1(G68R)-HZ screens that appeared in at least two screen replicates (Table 1). This has led to 50 SDL interactions that are dependent on the methyltransferase activity of Hmt1. The Venn diagram was generated using the online tool Venny *2.1* (https://bioinfogp.cnb.csic.es/tools/venny/).

Raw SDL data from eight replicate screens for WT Hmt1-HZ and eight replicate screens for Hmt1(G68R)-HZ and two replicate screens for HZ-tag are shown in Table S1. For each yeast strain, which was represented by quadruplicate colonies on the arrays, we generated the average growth and standard deviation (StdDev) in galactose (inducing) and glucose (non-inducing) conditions, as well as the calculated SDL score (growth in galactose/glucose) (Table S1). In total, 632 SDL interactions were generated from the eight replicate screens of WT Hmt1-HZ (Table S2), 622 SDL interactions from the eight replicate screens of Hmt1(G68R)-HZ (Table S3) and 53 SDL interactions from the two replicate screens of HZ-tag (Table S4). To eliminate random SDL interactions, we then selected those that appeared in at least two screen replicates resulting in 129 SDL interactions for WT Hmt1-HZ, 101 for Hmt1(G68R)-HZ and 15 for the HZ-tag (Table 1). Next, we filtered the SDL interactions of WT Hmt1-HZ by removing all interactions also found for Hmt1(G68R)-HZ and HZ-tag, in order to eliminate those that are independent of the Hmt1 methyltransferase activity and false-positive interactions respectively (Fig. 1). This filtering analysis resulted in 50 high-confidence SDL interactions of WT Hmt1-HZ that represent putative arginine methylation regulators and are listed along with their Gene Ontology (GO) Annotated Term in Table 2. GO enrichment analysis of the 50 filtered SDL interactions (Table S5) identified eight significantly enriched biological process terms, as shown in Table 3. Notably, Hmt1 (or its human ortholog PRMT1) has already been associated with some of the enriched biological processes like cytosolic transport, metabolism, and RNA processing [4], [5], [6], [7], [8].

**Table 1:** Number of total analysed interactions generated from all SDL screens.

SDL Screen	SDL Interactions
a pGAL-Hmt1-His_6_—HA-ZZ	
Replicate 1	122
Replicate 2	73
Replicate 3	93
Replicate 4	68
Replicate 5	124
Replicate 6	69
Replicate 7	44
Replicate 8	39
SDL Interactions appearing in at least two replicate screens:	**129**
b pGAL-Hmt1(G68R)-His_6_—HA-ZZ	
Replicate 1	104
Replicate 2	105
Replicate 3	88
Replicate 4	66
Replicate 5	108
Replicate 6	73
Replicate 7	42
Replicate 8	36
SDL Interactions appearing in at least two replicate screens:	**101**
c pHis_6_—HA-ZZ	
Replicate 1	29
Replicate 2	24
SDL Interactions appearing in at least two replicate screens:	**15**

*Note:*.

a plasmid overexpressing Hmt1 wild-type.

b plasmid overexpressing Hmt1(G68R) catalytically mutant.

c plasmid expressing only the His_6_—HA-ZZ tag.

**Table 2:** Filtered SDL interactions of Hmt1 shortlisted from all screens.

SDL Interactions			
Systematic Name	Standard Name	Gene Ontology (GO) Term (GO ID)	Gene Description
YBR181C	RPS6B	rRNA processing (GO:0,006,364)	Protein component of the small (40S) ribosomal subunit; homologous to mammalian ribosomal protein S6, no bacterial homolog; phosphorylated on S233 by Ypk3p in a TORC1-dependent manner, and on S232 in a TORC1/2-dependent manner by Ypk1/2/3p; RPS6B has a paralog, RPS6A, that arose from the whole genome duplication
YCL030C	HIS4	cellular amino acid metabolic process (GO:0,006,520)	Multifunctional enzyme containing phosphoribosyl-ATP pyrophosphatase, phosphoribosyl-AMP cyclohydrolase, and histidinol dehydrogenase activities; catalyzes the second, third, ninth and tenth steps in histidine biosynthesis
YCR053W	THR4	cellular amino acid metabolic process (GO:0,006,520)	Threonine synthase, conserved protein that catalyzes formation of threonine from O-phosphohomoserine; expression is regulated by the GCN4-mediated general amino acid control pathway
YDL159W	STE7	protein phosphorylation (GO:0,006,468)	Pheromone-responsive MAPK scaffold protein; couples activation of the G-protein-coupled pheromone receptor to MAPK activation; intramolecular interaction of PH and VWA domains blocks activation of assembled signalling cascade components (Ste11p, Ste7p and Fus3p) under basal conditions; Gbeta-gamma (Ste4p-Ste18p)-dependent docking at the plasma membrane and binding of PI(4,5)P2 by the PH domain relieves autoinhibition, resulting in pheromone-dependent pathway activation
YDR234W	LYS4	cellular amino acid metabolic process (GO:0,006,520)	Homoaconitase, catalyzes the conversion of homocitrate to homoisocitrate, which is a step in the lysine biosynthesis pathway
YEL060C	PRB1	sporulation (GO:0,043,934)	Vacuolar proteinase B (yscB) with H3 N-terminal endopeptidase activity; serine protease of the subtilisin family; involved in protein degradation in the vacuole and required for full protein degradation during sporulation; activity inhibited by Pbi2p; protein abundance increases in response to DNA replication stress; PRB1 has a paralog, YSP3, that arose from the whole genome duplication
YER083C	GET2	mitochondrion organization (GO:0,007,005)	Subunit of the GET complex; involved in insertion of proteins into the ER membrane; required for the retrieval of HDEL proteins from the Golgi to the ER in an ERD2 dependent fashion and for meiotic nuclear division
YFL013C	IES1	chromatin organization (GO:0,006,325)	Subunit of the INO80 chromatin remodelling complex; relocalizes to the cytosol in response to hypoxia
YGL105W	ARC1	tRNA aminoacylation for protein translation (GO:0,006,418)	Protein that binds tRNA and methionyl- and glutamyl-tRNA synthetases; involved in tRNA delivery, stimulating catalysis, and ensuring localization; also binds quadruplex nucleic acids; protein abundance increases in response to DNA replication stress; methionyl-tRNA synthetase is Mes1p; glutamyl-tRNA synthetase is Gus1p
YGL255W	ZRT1	transmembrane transport (GO:0,055,085)	High-affinity zinc transporter of the plasma membrane, responsible for the majority of zinc uptake; transcription is induced under low-zinc conditions by the Zap1p transcription factor
YGR122W	N/A	transcription by RNA polymerase II (GO:0,006,366)	Protein that may be involved in pH regulation; probable ortholog of A. nidulans PalC, which is involved in pH regulation and binds to the ESCRT-III complex; null mutant does not properly process Rim101p and has decreased resistance to rapamycin; GFP-fusion protein is cytoplasmic; relative distribution to cytoplasm increases upon DNA replication stress
YHR030C	SLT2	protein phosphorylation (GO:0,006,468)	Serine/threonine MAP kinase; coordinates expression of all 19S regulatory particle assembly-chaperones (RACs) to control proteasome abundance; involved in regulating maintenance of cell wall integrity, cell cycle progression, nuclear mRNA retention in heat shock, septum assembly; required for mitophagy, pexophagy; affects recruitment of mitochondria to phagophore assembly site; plays role in adaptive response of cells to cold; regulated by the PKC1-mediated signaling pathway
YIL090W	ICE2	organelle assembly (GO:0,070,925)	Integral ER membrane protein with type-III transmembrane domains; required for maintenance of ER zinc homeostasis; necessary for efficient targeting of Trm1p tRNA methyltransferase to inner nuclear membrane; mutations cause defects in cortical ER morphology in both the mother and daughter cells
YIR023W	DAL81	monocarboxylic acid metabolic process (GO:0,032,787)	Positive regulator of genes in multiple nitrogen degradation pathways; contains DNA binding domain but does not appear to bind the dodecanucleotide sequence present in the promoter region of many genes involved in allantoin catabolism
YJL101C	GSH1	cofactor metabolic process (GO:0,051,186)	Gamma glutamylcysteine synthetase; catalyzes the first step in glutathione (GSH) biosynthesis; expression induced by oxidants, cadmium, and mercury; protein abundance increases in response to DNA replication stress
YML013C-A	N/A	N/A	Dubious open reading frame; unlikely to encode a functional protein, based on available experimental and comparative sequence data; partially overlaps the verified gene SEL1
YML090W	N/A	N/A	Dubious open reading frame; unlikely to encode a functional protein, based on available experimental and comparative sequence data; partially overlaps the dubious ORF YML089C; exhibits growth defect on a non-fermentable (respiratory) carbon source
YNL227C	JJJ1	endocytosis (GO:0,006,897)	Co-chaperone that stimulates the ATPase activity of Ssa1p; required for a late step of ribosome biogenesis; associated with the cytosolic large ribosomal subunit; contains a J-domain; mutation causes defects in fluid-phase endocytosis
YOR069W	VPS5	endosomal transport (GO:0,016,197)	Nexin-1 homolog; required for localizing membrane proteins from a prevacuolar/late endosomal compartment back to late Golgi; structural component of retromer membrane coat complex; forms a retromer subcomplex with Vps17p; required for recruiting the retromer complex to the endosome membranes; VPS5 has a paralog, YKR078W, that arose from the whole genome duplication
YOR132W	VPS17	endosomal transport (GO:0,016,197)	Subunit of the membrane-associated retromer complex; essential for endosome-to-Golgi retrograde protein transport; peripheral membrane protein that assembles onto the membrane with Vps5p to promote vesicle formation; required for recruiting the retromer complex to the endosome membranes
YPL086C	ELP3	histone modification (GO:0,016,570)	Subunit of Elongator complex; Elongator is required for modification of wobble nucleosides in tRNA; exhibits histone acetyltransferase activity that is directed to histones H3 and H4; disruption confers resistance to K. lactis zymotoxin; human homolog ELP3 can partially complement yeast elp3 null mutant
YPL262W	FUM1	cellular respiration (GO:0,045,333)	Fumarase; converts fumaric acid to l-malic acid in the TCA cycle; cytosolic and mitochondrial distribution determined by the N-terminal targeting sequence, protein conformation, and status of glyoxylate shunt; phosphorylated in mitochondria
YPR070W	MED1	transcription by RNA polymerase II (GO:0,006,366)	Subunit of the RNA polymerase II mediator complex; associates with core polymerase subunits to form the RNA polymerase II holoenzyme; essential for transcriptional regulation
YDL077C	VAM6	regulation of organelle organization (GO:0,033,043)	Guanine nucleotide exchange factor for the GTPase Gtr1p; subunit of the HOPS endocytic tethering complex; vacuole membrane protein; functions as a Rab GTPase effector, interacting with both GTP- and GDP-bound conformations of Ypt7p; facilitates tethering and promotes membrane fusion events at the late endosome and vacuole; required for both membrane and protein trafficking; component of vacuole-mitochondrion contacts (vCLAMPs) important for lipid transfer between organelles
YDL160C	DHH1	regulation of organelle organization (GO:0,033,043)	Cytoplasmic DEAD-box helicase, stimulates mRNA decapping; coordinates distinct steps in mRNA function and decay, interacting with both decapping and deadenylase complexes; role in translational repression, mRNA decay, and possibly mRNA export; interacts and cooperates with Ngr1p to promote specific mRNA decay; ATP- and RNA-bound form promotes processing body (PB) assembly, while ATPase stimulation by Not1p promotes PB disassembly; forms cytoplasmic foci on replication stress
YDR375C	BCS1	transmembrane transport (GO:0,055,085)	Protein translocase and chaperone required for Complex III assembly; member of the AAA ATPase family; forms a homo-oligomeric complex in the mitochondrial inner membrane that translocates the C-terminal domain of Rip1p from the matrix across the inner membrane and delivers it to an assembly intermediate of respiratory Complex III; also required for assembly of the Qcr10p subunit; mutation is functionally complemented by human homolog BCS1L, linked to neonatal diseases
YHR018C	ARG4	cellular amino acid metabolic process (GO:0,006,520)	Argininosuccinate lyase; catalyzes the final step in the arginine biosynthesis pathway
YIL020C	HIS6	cellular amino acid metabolic process (GO:0,006,520)	Enzyme that catalyzes the fourth step in the histidine pathway; Phosphoribosylformimino-5-aminoimidazole carboxamide ribotide isomerase; mutations cause histidine auxotrophy and sensitivity to Cu, Co, and Ni salts
YJL029C	VPS53	endosomal transport (GO:0,016,197)	Component of the GARP (Golgi-associated retrograde protein) complex; GARP is required for the recycling of proteins from endosomes to the late Golgi, and for mitosis after DNA damage induced checkpoint arrest; required for vacuolar protein sorting; members of the GARP complex are Vps51p-Vps52p-Vps53p-Vps54p; human ortholog is implicated in progressive cerebello-cerebral atrophy type 2 (PCCA2)
YML001W	YPT7	endosomal transport (GO:0,016,197)	Rab family GTPase; GTP-binding protein of the rab family; required for homotypic fusion event in vacuole inheritance, for endosome-endosome fusion; localizes to sites of contact between the vacuole and mitochondria (vCLAMPs); interacts with the cargo selection/retromer complex for retrograde sorting; similar to mammalian Rab7
YML106W	URA5	nucleobase-containing small molecule metabolic process (GO:0,055,086)	Major orotate phosphoribosyltransferase (OPRTase) isozyme; catalyzes the fifth enzymatic step in de novo biosynthesis of pyrimidines, converting orotate into orotidine-5′-phosphate; URA5 has a paralog, URA10, that arose from the whole genome duplication
YMR021C	MAC1	transcription by RNA polymerase II (GO:0,006,366)	Copper-sensing transcription factor; involved in regulation of genes required for high affinity copper transport; required for regulation of yeast copper genes in response to DNA-damaging agents; undergoes changes in redox state in response to changing levels of copper or MMS
YMR154C	RIM13	protein maturation (GO:0,051,604)	Calpain-like cysteine protease; involved in proteolytic activation of Rim101p in response to alkaline pH; localizes to punctate structures in alkaline conditions and in vps4 mutant; has similarity to A. nidulans palB
YOR068C	VAM10	vacuole organization (GO:0,007,033)	Protein involved in vacuole morphogenesis; acts at an early step of homotypic vacuole fusion that is required for vacuole tethering
YBR131W	CCZ1	protein targeting (GO:0,006,605)	Subunit of a heterodimeric guanine nucleotide exchange factor (GEF); subunit of the Mon1-Ccz1 GEF complex, which stimulates nucleotide exchange and activation of Ypt7p, a Rab family GTPase involved in membrane tethering and fusion events at the late endosome and vacuole; GEF activity is stimulated by membrane association and anionic phospholipids; involved in localizing Ypt7p to the vacuolar membrane; required for macroautophagy, the CVT pathway and mitophagy
YDL090C	RAM1	transferase activity (GO:0,016,740)	Beta subunit of the CAAX farnesyltransferase (FTase); this complex prenylates the a-factor mating pheromone and Ras proteins; required for the membrane localization of Ras proteins and a-factor; homolog of the mammalian FTase beta subunit
YLR015W	BRE2	histone modification (GO:0,016,570)	Subunit of COMPASS (Set1C) complex; COMPASS methylates Lys4 of histone H3 and functions in silencing at telomeres; has a C-terminal Sdc1 Dpy-30 Interaction (SDI) domain that mediates binding to Sdc1p; similar to trithorax-group protein ASH2L
YLR056W	ERG3	lipid metabolic process (GO:0,006,629)	C-5 sterol desaturase; glycoprotein that catalyzes the introduction of a C-5(6) double bond into episterol, a precursor in ergosterol biosynthesis; transcriptionally down-regulated when ergosterol is in excess; mutants are viable, but cannot grow on non-fermentable carbon sources; substrate of HRD ubiquitin ligase; mutation is functionally complemented by human SC5D
YLR262C	TMA7	endosomal transport (GO:0,016,197)	Protein of unknown that associates with ribosomes; null mutant exhibits translation defects, altered polyribosome profiles, and resistance to the translation inhibitor anisomcyin; protein abundance increases in response to DNA replication stress
YLR318W	EST2	telomere organization (GO:0,032,200)	Reverse transcriptase subunit of the telomerase holoenzyme; essential for telomerase core catalytic activity, involved in other aspects of telomerase assembly and function; mutations in human homolog are associated with aplastic anemia
YLR357W	RSC2	chromosome segregation (GO:0,007,059)	Component of the RSC chromatin remodeling complex; required for expression of mid-late sporulation-specific genes; involved in telomere maintenance; RSC2 has a paralog, RSC1, that arose from the whole genome duplication
YLR402W	N/A	N/A	Dubious open reading frame; unlikely to encode a functional protein, based on available experimental and comparative sequence data
YML121W	GTR1	response to starvation (GO:0,042,594)	Subunit of a TORC1-stimulating GTPase and the EGO/GSE complex; subunit of Gtr1-Gtr2, a GTPase that activates TORC1 in response to amino acid stimulation; subunit of EGO/GSE, a vacuolar/endosomal membrane complex that regulates exit from rapamycin-induced growth arrest and sorting of Gap1p from the endosome to the plasma membrane; involved in phosphate transport and telomeric chromatin silencing; activated by the Iml1p (GAP) subunit of the SEACIT complex; similar to human RagA and RagB
YMR116C	ASC1	response to chemical (GO:0,042,221)	G-protein beta subunit and guanine dissociation inhibitor for Gpa2p; ortholog of RACK1 that inhibits translation; core component of the small (40S) ribosomal subunit; required to prevent frameshifting at ribosomes stalled at repeated CGA codons; regulates P-body formation induced by replication stress; represses Gcn4p in the absence of amino acid starvation; controls phosphorylation of multiple proteins
YNL120C	N/A	N/A	Dubious open reading frame; unlikely to encode a functional protein, based on available experimental and comparative sequence data; deletion enhances replication of Brome mosaic virus in S. cerevisiae, but likely due to effects on the overlapping gene
YOR026W	BUB3	regulation of organelle organization (GO:0,033,043)	Kinetochore checkpoint WD40 repeat protein; localizes to kinetochores during prophase and metaphase, delays anaphase in the presence of unattached kinetochores; forms complexes with Mad1p-Bub1p and with Cdc20p, binds Mad2p and Mad3p; functions at kinetochore to activate APC/C—Cdc20p for normal mitotic progression
YOR070C	GYP1	endomembrane system (GO:0,012,505)	Cis-golgi GTPase-activating protein (GAP) for yeast Rabs; the Rab family members are Ypt1p (in vivo) and for Ypt1p, Sec4p, Ypt7p, and Ypt51p (in vitro); involved in vesicle docking and fusion; interacts with autophagosome component Atg8p
YJR077C	MIR1	transmembrane transport (GO:0,055,085)	Mitochondrial phosphate carrier; imports inorganic phosphate into mitochondria; functionally redundant with Pic2p but more abundant than Pic2p under normal conditions; phosphorylated
YLR442C	SIR3	regulation of organelle organization (GO:0,033,043)	Silencing protein; interacts with Sir2p, Sir4p, and histone H3/H4 tails to establish transcriptionally silent chromatin; required for spreading of silenced chromatin; recruited to chromatin through interaction with Rap1p; C-terminus assumes variant winged helix-turn-helix (wH) fold that mediates homodimerization, which is critical for holo-SIR complex loading; required for telomere hypercluster formation in quiescent yeast cells; has paralog ORC1 from whole genome duplication
YML097C	VPS9	vacuole organization (GO:0,007,033)	Guanine nucleotide exchange factor (GEF) and ubiquitin receptor; involved in vesicle-mediated vacuolar transport, including Golgi-endosome trafficking and sorting through the multivesicular body (MVB); stimulates the intrinsic guanine nucleotide exchange activity of Rab family members (Vps21p/Ypt52p/Ypt53p); partially redundant with GEF MUK1; required for localization of the CORVET complex to endosomes; similar to mammalian ras inhibitors; contains a Ub-interacting CUE domain

**Table 3:** Significantly enriched Gene Ontology (GO) biological process terms represented within the Hmt1 SDL interactions.

GO Term	Systematic gene names included	*p*-value (<0,05)
endosomal transport (GO:0,016,197)	YJL029C, YLR262C, YML001W, YML097C, YOR069W, YOR132W	0.0004
vacuole organization (GO:0,007,033)	YDL077C, YLR262C, YML001W, ML097C, YOR068C	0.0028
organelle inheritance (GO:0,048,308)	YIL090W, YML001W, YML097C	0.0139
cellular amino acid metabolic process (GO:0,006,520)	YCL030C, YCR053W, YDR234W, YHR018C, YIL020C, YIR023W	0.0207
RNA catabolic process (GO:0,006,401)	YDL160C, YGR122W, YLR442C, YMR116C	0.0262
organelle fusion (GO:0,048,284)	YDL077C, YML001W, YOR068C	0.0382
regulation of organelle organization (GO:0,033,043)	YDL077C, YDL160C, YLR442C, YML001W, YOR026W	0.0468
translational elongation (GO:0,006,414)	YDL160C, YMR116C	0.0491

## 2 Experimental design, materials, and methods

### 2.1 SDL rationale for this study

**Fig. 2:**
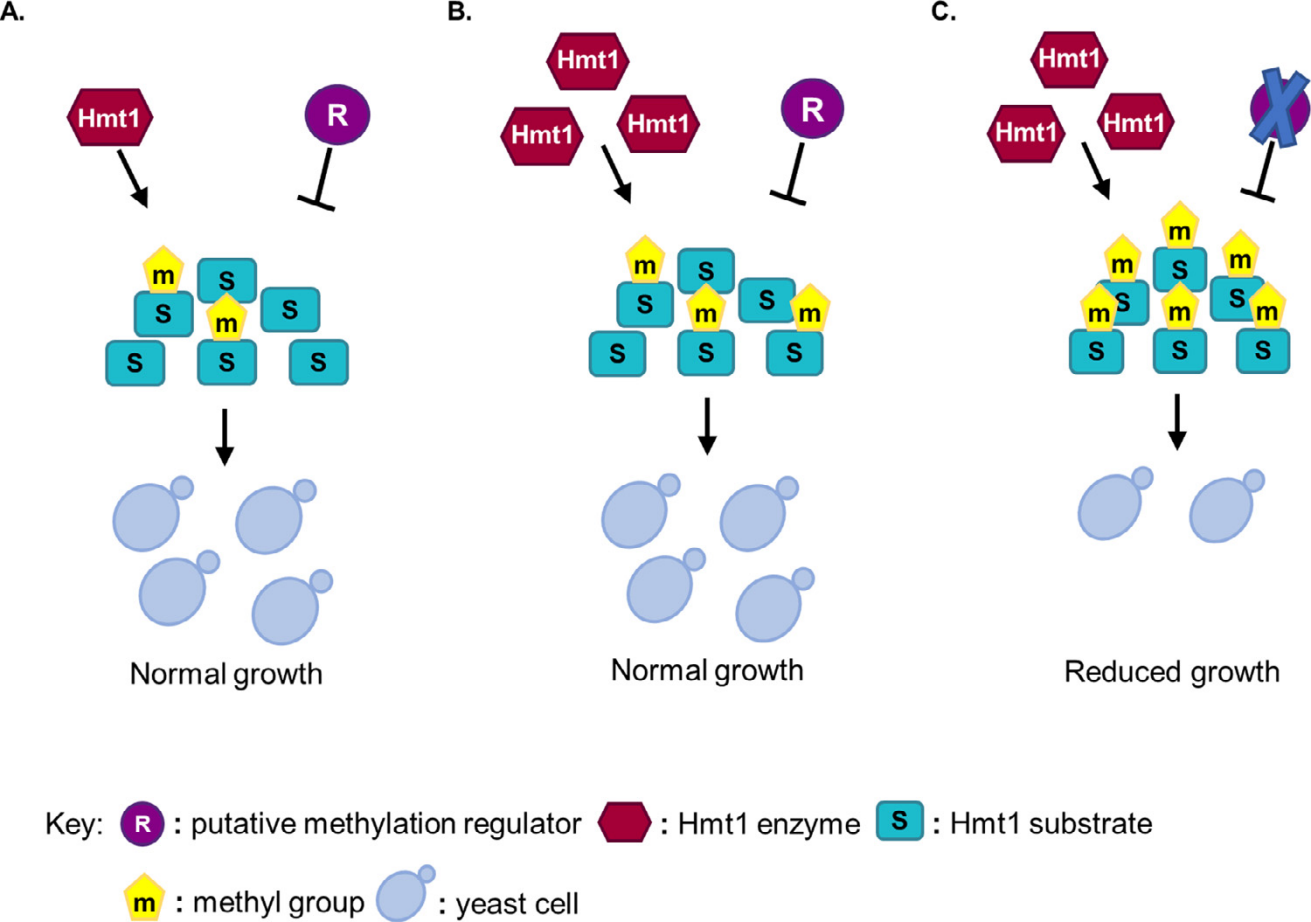
**Rationale of synthetic dosage lethality (SDL) for the identification of putative arginine methylation regulators. (A)** The presence of physiological levels of Hmt1 arginine methyltransferase and arginine methylation regulator (R) leads to normal growth. This putative methylation regulator could control the activity of the Hmt1 enzyme or methylation of its substrate(s) or any other step during the methylation/demethylation process. **(B)** Overexpression of Hmt1 arginine methyltransferase does not lead to a severe phenotype as its activity is antagonised by an intact regulator (demethylase, PRMT-regulator, modification crosstalk). General levels of arginine methylation on substrates (S) are maintained at physiological levels or increased at a tolerable level for the cells. **(C)** Combination of Hmt1 methyltransferase overexpression with deletion or inactivation of an arginine methylation regulator (R) results in reduced growth due to toxic hypermethylation of substrate(s). At least 30% reduction in growth would indicate an SDL interaction between Hmt1 and the putative regulator.

The rationale supporting the performed SDL screens is based on the observation that the phenotype resulting from an overexpressing protein is exacerbated by deleting a second gene [10]. Particularly, overexpressing Hmt1 alone does not lead to lethality or growth reduction compared to endogenous basal expression (Fig. 2A) because arginine methylation regulators (i.e. demethylase, PRTM-regulators, modification crosstalk) are still intact to limit the levels of arginine methylation on Hmt1 substrates (Fig. 2B). In contrast, overexpression of Hmt1 in combination with the deletion of an arginine methylation regulator will result in toxic hypermethylation of substrates, which cannot be tolerated by cells and therefore causes a growth defect (Fig. 2C). Hence, the arising objective was to screen the entire yeast genome for genes whose deletion leads to severe growth defect when the predominant arginine methyltransferase Hmt1 is overexpressed.

### 2.2 Yeast strains and plasmid construction

**Fig. 3:**
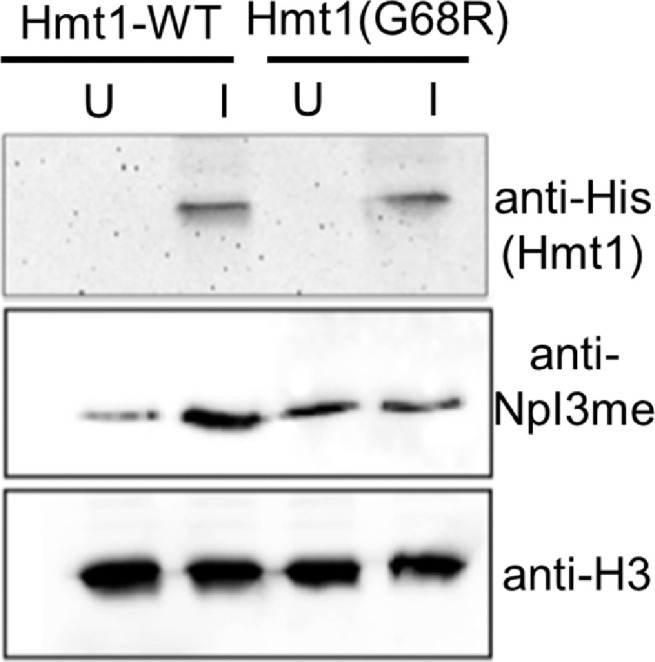
**Validation of overexpression of exogenous proteins from constructs and verification of reduced methylase activity of Hmt1(G68R).** Immunoblot analysis of extracts isolated from strains grown in uninduced (*U* = glucose) and induced (*I* =galactose) conditions, to confirm overexpression for Hmt1 wild-type and catalytically mutant Hmt1(G68R). Overexpression of WT Hmt1-HZ in induced conditions results in increased methylated levels of its known substrate NpI3. Upon overexpression of Hmt1(G68R)-HZ, the methylation levels of NpI3 are unchanged between uninduced and induced conditions. The detected methylation of Npl3 in the Hmt1(G68R)-HZ strains is mediated by the endogenous wild-type Hmt1 enzyme. The levels of overexpressed Hmt1 were detected using an anti-His antibody recognizing the HZ tag and of its substrate Npl3 was detected using an anti-Npl3 methylated antibody. The loading of protein extracts was monitored by an anti-histone H3 antibody.

For the construction of inducible vectors, a multicopy MORF (moveable ORF) plasmid was used, which contained a URA3 selectable marker, a HIS_6_—HA-ZZ (HZ) tag and *PGAL,* a galactose inducible promoter of the galactokinase gene *GAL1,* that overexpressed either *HMT1* gene with the complete wild type sequence (Hmt1-WT) or *hmt1* gene with the catalytic mutant sequence (Hmt1-G68R) (Fig. 3) [4]. A third empty MORF vector that expressed only the HZ tag was used as a control. The query strain Y7092 (*MATα can1*∆::*STE2pr-his5 lyp1∆ ura3∆0 leu2∆0 his3∆1 met15∆0*) was transformed separately with all three inducible vectors and mated to the deletion mutant array (DMA) library consisting of 4548 BY4741 (*MΑΤa* CAN1 LYP1 *genex*Δ::kanMX) single knockout strains with each one carrying deletion of a non-essential gene [11]. The deleted genes were replaced with the antibiotic marker KanMX4, which confers resistance to Geneticin (G418).

### 2.3 SDL screen data collection

**Fig. 4:**
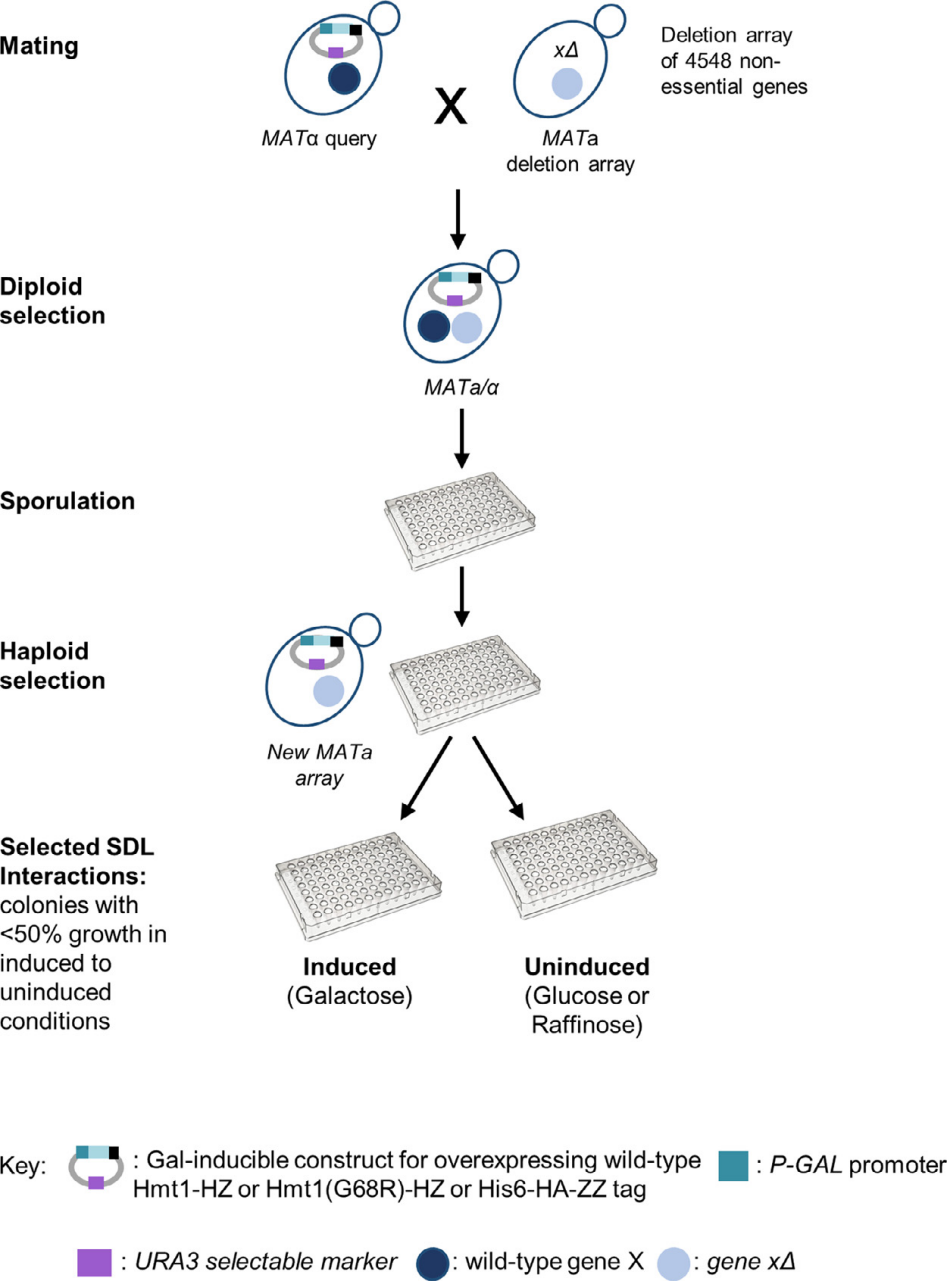
**Construction of SDL arrays and collection of SDL Interaction data.** Schematic representation of the SDL procedure for the identification of SDL interactions. Haploid cells carrying an overexpressing plasmid of 1) wild-type Hmt1-HZ, or 2) Hmt1(G68R)-HZ, or 3) the HZ (His_6_—HA-ZZ) tag were mated to an array of 4548 haploid cells with individual gene deletions. After several steps of replica-pinning and selection, an array of haploid cells was isolated that contained the overexpressing construct in combination with a specific gene deletion. The array was then grown on un-induced (glucose or raffinose) and induced (galactose) conditions to identify cells that would show reduced growth phenotype when Hmt1 is overexpressed in a specific gene deletion background.

An outline of the SDL procedure performed during this study is indicated in Fig. 4. The initial DMA library, which consists of 14 array plates was replicated by the BM3-BC automated pinning robot on solid (2% agar) rich medium plates with 2% glucose (YPAD) and the addition of G418. The plates were incubated at 30 °C for 2 days. Four lawn plates of the control strain and four of each query strain were prepared. Each strain was incubated overnight in 5 ml Synthetic Complete liquid medium without uracil (SC-URA) and with 2% glucose at 30 °C. The next day, 800μl of culture were spread on SC-URA lawn plates and the plates were incubated at room temperature (RT) for 3 days. For the mating process, the query strain was pinned from the lawn plates onto fresh YPAD mating plates, followed by pinning of the DMA library on top of the query cells (Fig. 4). The plates were incubated for 3 days in RT. Selection of diploid cells first involved pinning from the mating plates onto the diploid plates (SC-URA), followed by 2 days incubation at 30 °C. Then, cells from diploid plates were pinned onto SC plates with the addition of G418 and incubated for 2 days at 30 °C. After diploid selection, cells were pinned on enriched solid sporulation medium (reduced carbon and nitrogen levels) and incubated for 7 days in RT for the induction of sporulation and the formation of haploid meiotic spore progeny (Fig. 4). The formed spores were next pinned onto solid Synthetic Defined medium without histidine, arginine, lysine and uracil and with the addition of canavanine and thialysine (SD – His/Arg/Lys/URA+canavanine/thialysine), for the selection of the *MATa* haploid meiotic progeny. Plates were incubated for 2 days at 30 °C, and the haploid selection step was repeated twice. As a prefinal step, cells were pinned on SD – His/Arg/Lys/URA+canavanine/thialysine plates with the addition of G418, for selection of the meiotic progeny that carries the gene deletion mutation (*genexΔ*::kanMX) and incubated for 2 days at 30 °C. SDL interactions were selected by pinning the constructed cells onto SC plates, either containing 2% glucose or raffinose as non-inductive conditions, or 2% galactose as inductive conditions (Fig. 4). The SDL interactions haploid selection step was repeated 8 times with the query strain carrying the plasmid overexpressing WT Hmt1-HZ and 8 times with the query strain carrying the plasmid overexpressing Hmt1(G68R)-HZ (Table 1). It was additionally assessed 2 times with the query strain carrying the empty vector expressing the HZ tag only, as a control (Table 1). Each plate was digitally photographed and the relative growth of individual colonies was processed with RobosoftPro application, that quantifies the pixels of any given colony [3]. Comparison between average growth of the technical quadruplicate colonies representing each strain in non-inducing conditions, with the corresponding four colonies of the strain in inducing conditions was generated as standard deviation (StdDev) by the application (Table S1). An SDL score indicating the overall growth observed was calculated by the equation (WT Hmt1-HZ Galactose/Glucose) (Table S1).

### 2.4 Data analysis and filtering

The raw data of SDL screens were subjected to initial analysis by following three steps: 1) Removal of YOR202W gene values which were located in the outermost two lines of each plate. These colonies represent a border control in order to avoid irregular growth of strains positioned around the edges of the plate and thus serve as an internal quality control for each screen. 2) Removal of strains with <=60% growth on glucose compared to the average growth of all the other strains on the plate, in order to exclude strains exhibiting severe growth defect even in uninduced conditions. 3) Selection of hits with SDL score (log10≤ −0,15) ≤ 70%, meaning that they have at least 30% growth defect (threshold set by [3]) when grown on galactose compared to glucose. The SDL interactions were further filtered by executing six rigorous selection steps: 1) removal of SDL interactions giving SDL scores >70% in other screen replicates, 2) removal of SDL interactions generated only once among all screen replicates, 3) removal of all galactose-induced genes, 4) removal of genes which displayed significant growth changes when comparing all screens under non-inducible (glucose) conditions, 5) removal of 15 SDL interactions generated by the HZ-tag screen, and 6) removal of 102 SDL interactions generated by the Hmt1(G68R)-HZ screen.

### 2.5 Gene ontology (GO) enrichment analysis

The filtered genes (Table 2) corresponding to the high-confidence Hmt1 SDL interactions identified through the screens were clustered into their annotated GO biological processes according to the Saccharomyces Genome Database (SGD) GO Slim Mapper (https://www.yeastgenome.org/goSlimMapper) (Table S5). The Fisher's exact test was used to classify statistically significant enriched GO terms (*p*-value ≤ 0,05) (Table 3).

## CRediT authorship contribution statement

**Dimitris Kyriakou:** Methodology, Validation, Formal analysis, Investigation, Visualization, Writing - review & editing. **Mamantia Constantinou:** Data curation, Visualization, Writing - original draft. **Antonis Kirmizis:** Conceptualization, Supervision, Project administration, Funding acquisition, Writing - review & editing.

## Declaration of Competing Interest

DK was employed by company EFEVRE TECH LTD. All authors declare that they have no known competing financial interests or personal relationships which have, or could be perceived to have, influenced or have been influenced by the work reported in this article.

## Appendix Supplementary materials

Image, application 1

Image, application 2

Image, application 3

Image, application 4
