# Supplementary material for: Synthetic dosage lethal (SDL) interaction data of Hmt1 arginine methyltransferase
Source: Data Brief. 2020 Jun 21;31:105885. doi: 10.1016/j.dib.2020.105885 (PMC7330151; doi:10.1016/j.dib.2020.105885)
Supplement: Supplementary file 3 [file mmc3.docx]

| Table S2. Analysed SDL Interactions from all replicates of Hmt1-WT SDL screens | | | | | | | |
| --- | --- | --- | --- | --- | --- | --- | --- |
| O/E Hmt1 WT Rep.1 | **O/E Hmt1 WT Rep.2** | **O/E Hmt1 WT Rep.3** | **O/E Hmt1 WT Rep.4** | **O/E Hmt1 WT Rep.5** | **O/E Hmt1 WT Rep.6** | **O/E Hmt1 WT Rep.7** | **O/E Hmt1 WT Rep.8** |
| YBL031W | YBL016W | YBR018C | YBL072C | YAR003W | YBR018C | YBR018C | YBR018C |
| YBR018C | YBR018C | YBR019C | YBR018C | YBR018C | YBR019C | YBR019C | YBR019C |
| YBR019C | YBR019C | YBR020W | YBR019C | YBR019C | YBR020W | YBR020W | YBR020W |
| YBR020W | YBR020W | YBR020W | YBR020W | YBR020W | YBR020W | YBR020W | YBR020W |
| YBR020W | YBR020W | YBR026C | YBR020W | YBR020W | YBR115C | YBR248C | YBR248C |
| YBR026C | YBR131W | YCL037C | YBR093C | YBR026C | YBR131W | YCR071C | YCR071C |
| YBR106W | YBR181C | YCR009C | YBR181C | YBR131W | YBR248C | YDL006W | YDL006W |
| YBR131W | YBR267W | YCR028C-A | YBR267W | YBR175W | YCL030C | YDL090C | YDL090C |
| YBR267W | YCR009C | YCR071C | YBR289W | YBR248C | YCL032W | YDL159W | YDL159W |
| YCR009C | YCR028C-A | YCR081W | YCL032W | YCL030C | YCR009C | YDR009W | YDR009W |
| YCR028C-A | YCR053W | YDL002C | YCR009C | YCL032W | YCR028C-A | YEL013W | YEL013W |
| YCR071C | YCR071C | YDL006W | YCR028C-A | YCR009C | YCR071C | YER061C | YER153C |
| YCR081W | YCR081W | YDL074C | YCR053W | YCR028C-A | YDL006W | YER153C | YFL036W |
| YDL002C | YDL160C | YDL160C | YCR071C | YCR071C | YDL090C | YFL036W | YFR025C |
| YDL006W | YDR009W | YDR009W | YCR081W | YCR081W | YDR009W | YFR025C | YGL024W |
| YDL033C | YDR034C | YDR049W | YDL041W | YDL002C | YDR034C | YGL105W | YGL255W |
| YDL074C | YDR290W | YDR162C | YDL160C | YDL006W | YDR127W | YGR183C | YHR116W |
| YDL074C | YDR316W | YDR290W | YDR009W | YDL074C | YDR162C | YHR067W | YIL020C |
| YDL077C | YDR388W | YDR375C | YDR127W | YDL074C | YDR234W | YHR116W | YJL071W |
| YDR009W | YEL013W | YDR388W | YDR290W | YDL077C | YDR290W | YJR077C | YJR077C |
| YDR049W | YEL062W | YEL013W | YDR378C | YDL090C | YDR316W | YJR090C | YJR090C |
| YDR074W | YER153C | YEL060C | YDR388W | YDR009W | YDR388W | YLR038C | YLR038C |
| YDR080W | YFL036W | YER061C | YEL013W | YDR049W | YEL013W | YLR218C | YLR218C |
| YDR162C | YGL024W | YER083C | YEL060C | YDR127W | YER055C | YLR239C | YLR239C |
| YDR207C | YHR116W | YER153C | YEL062W | YDR148C | YER151C | YLR318W | YLR318W |
| YDR290W | YHR199C | YFL036W | YEL063C | YDR162C | YER153C | YLR337C | YLR337C |
| YDR316W | YIL116W | YGL024W | YER153C | YDR234W | YFL036W | YLR420W | YLR357W |
| YDR375C | YJL071W | YGL045W | YFL036W | YDR289C | YFR025C | YLR442C | YLR420W |
| YDR388W | YJL088W | YGL046W | YGL026C | YDR290W | YGL024W | YML106W | YLR442C |
| YDR512C | YJR090C | YGL167C | YGL255W | YDR316W | YGL154C | YMR282C | YML106W |
| YEL013W | YKL184W | YGL168W | YHR005C | YDR375C | YGL219C | YNL046W | YMR282C |
| YER061C | YKL212W | YGL219C | YHR018C | YDR388W | YGR078C | YOL009C | YNL046W |
| YER092W | YKL216W | YGL244W | YHR081W | YEL013W | YGR105W | YOL058W | YOL009C |
| YER151C | YLL044W | YGR101W | YHR116W | YER061C | YGR188C | YOR065W | YOR065W |
| YER153C | YLR015W | YGR105W | YIL090W | YER083C | YHR018C | YOR196C | YOR202W |
| YFL013C | YLR038C | YGR183C | YJR077C | YER092W | YHR116W | YOR202W | YPL172C |
| YFL036W | YLR039C | YHR067W | YKL212W | YER151C | YIL020C | YOR221C | YPL248C |
| YGL024W | YLR056W | YHR116W | YLR038C | YER153C | YIL090W | YPL172C | YPR024W |
| YGL045W | YLR061W | YIR023W | YLR056W | YFL013C | YJL071W | YPL180W | YPR191W |
| YGL046W | YLR081W | YJL029C | YLR061W | YFL036W | YJL088W | YPL226W |  |
| YGL105W | YLR182W | YJL046W | YLR081W | YFR025C | YJL204C | YPL248C |  |
| YGL167C | YLR192C | YJL204C | YLR182W | YGL023C | YJR090C | YPL262W |  |
| YGL168W | YLR218C | YJR077C | YLR218C | YGL024W | YLR038C | YPR134W |  |
| YGL212W | YLR261C | YKL184W | YLR337C | YGL045W | YLR081W | YPR191W |  |
| YGL219C | YLR262C | YKL212W | YLR338W | YGL046W | YLR218C |  |  |
| YGR105W | YLR318W | YLR015W | YLR357W | YGL149W | YLR337C |  |  |
| YGR122W | YLR337C | YLR038C | YLR442C | YGL154C | YLR338W |  |  |
| YGR183C | YLR338W | YLR039C | YML048W | YGL167C | YLR442C |  |  |
| YHR030C | YLR357W | YLR056W | YML063W | YGL168W | YML048W |  |  |
| YHR067W | YLR402W | YLR061W | YMR269W | YGL219C | YML097C |  |  |
| YHR116W | YLR420W | YLR081W | YMR282C | YGL244W | YMR165C |  |  |
| YHR189W | YML001W | YLR182W | YNL021W | YGR105W | YMR269W |  |  |
| YIR023W | YML013C-A | YLR192C | YNL120C | YGR122W | YMR282C |  |  |
| YJL029C | YML048W | YLR218C | YNL227C | YHL027W | YNL021W |  |  |
| YJL071W | YML063W | YLR239C | YOL001W | YHR012W | YNL120C |  |  |
| YJL101C | YML097C | YLR337C | YOL009C | YHR018C | YOL001W |  |  |
| YJL204C | YML106W | YLR338W | YOR065W | YHR030C | YOL009C |  |  |
| YJR077C | YML121W | YLR357W | YOR078W | YHR067W | YOR065W |  |  |
| YJR090C | YMR021C | YML001W | YOR293W | YHR111W | YOR070C |  |  |
| YKL184W | YMR063W | YML048W | YPL172C | YHR116W | YPL172C |  |  |
| YKL204W | YMR105C | YML063W | YPL180W | YIL008W | YPL213W |  |  |
| YKL212W | YMR116C | YML097C | YPL213W | YIL020C | YPL226W |  |  |
| YLR015W | YMR269W | YML121W | YPL226W | YJL029C | YPL248C |  |  |
| YLR038C | YMR282C | YMR063W | YPL248C | YJL101C | YPR024W |  |  |
| YLR039C | YNL064C | YMR116C | YPR024W | YJL154C | YPR044C |  |  |
| YLR056W | YNL236W | YMR154C | YPR134W | YJL204C | YPR134W |  |  |
| YLR061W | YNR010W | YMR269W | YPR179C | YJR073C | YPR173C |  |  |
| YLR081W | YOL009C | YMR282C |  | YKL087C | YPR179C |  |  |
| YLR182W | YOL081W | YNL021W |  | YKL184W |  |  |  |
| YLR192C | YPL172C | YNL227C |  | YKR007W |  |  |  |
| YLR218C | YPL248C | YNL273W |  | YLR015W |  |  |  |
| YLR239C | YPR173C | YNL302C |  | YLR038C |  |  |  |
| YLR261C | YPR179C | YOL001W |  | YLR081W |  |  |  |
| YLR262C |  | YOL009C |  | YLR192C |  |  |  |
| YLR318W |  | YOL108C |  | YLR218C |  |  |  |
| YLR337C |  | YOR026W |  | YLR239C |  |  |  |
| YLR393W |  | YOR030W |  | YLR337C |  |  |  |
| YLR402W |  | YOR065W |  | YLR393W |  |  |  |
| YML001W |  | YOR068C |  | YLR442C |  |  |  |
| YML013C-A |  | YOR069W |  | YML048W |  |  |  |
| YML048W |  | YOR070C |  | YML090W |  |  |  |
| YML090W |  | YOR196C |  | YML097C |  |  |  |
| YML097C |  | YOR221C |  | YML121W |  |  |  |
| YML121W |  | YOR275C |  | YMR021C |  |  |  |
| YMR021C |  | YOR334W |  | YMR063W |  |  |  |
| YMR063W |  | YPL086C |  | YMR116C |  |  |  |
| YMR105C |  | YPL172C |  | YMR154C |  |  |  |
| YMR116C |  | YPL226W |  | YMR165C |  |  |  |
| YMR154C |  | YPL248C |  | YMR282C |  |  |  |
| YMR269W |  | YPR134W |  | YMR312W |  |  |  |
| YMR282C |  | YPR173C |  | YNL021W |  |  |  |
| YNL021W |  | YPR179C |  | YNL052W |  |  |  |
| YNL081C |  | YPR191W |  | YNL080C |  |  |  |
| YNL119W |  |  |  | YNL081C |  |  |  |
| YNL120C |  |  |  | YNL119W |  |  |  |
| YNL197C |  |  |  | YNL120C |  |  |  |
| YNL236W |  |  |  | YNL169C |  |  |  |
| YNL265C |  |  |  | YNL197C |  |  |  |
| YNR006W |  |  |  | YNL215W |  |  |  |
| YNR010W |  |  |  | YOL001W |  |  |  |
| YOL009C |  |  |  | YOL009C |  |  |  |
| YOL108C |  |  |  | YOL012C |  |  |  |
| YOR026W |  |  |  | YOL108C |  |  |  |
| YOR030W |  |  |  | YOR030W |  |  |  |
| YOR065W |  |  |  | YOR065W |  |  |  |
| YOR068C |  |  |  | YOR068C |  |  |  |
| YOR069W |  |  |  | YOR070C |  |  |  |
| YOR070C |  |  |  | YOR132W |  |  |  |
| YOR106W |  |  |  | YOR196C |  |  |  |
| YOR132W |  |  |  | YOR202W |  |  |  |
| YOR196C |  |  |  | YOR221C |  |  |  |
| YOR275C |  |  |  | YOR275C |  |  |  |
| YOR309C |  |  |  | YOR334W |  |  |  |
| YPL172C |  |  |  | YPL055C |  |  |  |
| YPL226W |  |  |  | YPL060W |  |  |  |
| YPL248C |  |  |  | YPL086C |  |  |  |
| YPR024W |  |  |  | YPL172C |  |  |  |
| YPR070W |  |  |  | YPL226W |  |  |  |
| YPR134W |  |  |  | YPL248C |  |  |  |
| YPR173C |  |  |  | YPR070W |  |  |  |
| YPR179C |  |  |  | YPR134W |  |  |  |
| YPR191W |  |  |  | YPR173C |  |  |  |
|  |  |  |  | YPR179C |  |  |  |
|  |  |  |  | YPR191W |  |  |  |

| Table S3. Analysed SDL Interactions from all replicates of Hmt1(G68R) screens | | | | | | | |
| --- | --- | --- | --- | --- | --- | --- | --- |
| O/E Hmt1 cat.mut Rep.1 | **O/E Hmt1 cat.mut Rep.2** | **O/E Hmt1 cat.mut**  **Rep.3** | **O/E Hmt1**  **cat.mut**  **Rep.4** | **O/E Hmt1**  **cat.mut Rep.5** | **O/E Hmt1**  **cat.mut Rep.6** | **O/E Hmt1**  **cat.mut Rep.7** | **O/E Hmt1**  **cat.mut**  **Rep.8** |
| YBR020W | YBR019C | YBR018C | YBR018C | YAR003W | YBR018C | YBR018C | YBR018C |
| YCL037C | YBR020W | YBR019C | YBR019C | YBR018C | YBR019C | YBR019C | YBR019C |
| YCR009C | YBR267W | YBR020W | YBR020W | YBR019C | YBR020W | YBR020W | YBR020W |
| YCR028C-A | YCL030C | YBR020W | YBR020W | YBR020W | YBR020W | YBR020W | YBR020W |
| YCR071C | YCL032W | YBR106W | YBR093C | YBR020W | YBR106W | YBR248C | YBR248C |
| YCR081W | YCR009C | YBR200W | YBR200W | YBR026C | YBR131W | YBR289W | YBR289W |
| YDL002C | YCR028C-A | YCR009C | YBR289W | YBR131W | YBR248C | YCR028C-A | YCR028C-A |
| YDL006W | YCR053W | YCR028C-A | YCR009C | YBR175W | YBR267W | YCR071C | YCR071C |
| YDL033C | YCR071C | YCR071C | YCR028C-A | YCL032W | YCL030C | YDL006W | YDR034C |
| YDL074C | YCR081W | YCR081W | YCR053W | YCR009C | YCL032W | YDR316W | YDR227W |
| YDL074C | YDR009W | YDL002C | YCR071C | YCR028C-A | YCR009C | YDR410C | YDR316W |
| YDR009W | YDR034C | YDL006W | YCR081W | YCR071C | YCR028C-A | YER153C | YER153C |
| YDR049W | YDR049W | YDL074C | YDL006W | YCR081W | YCR071C | YFL036W | YFR025C |
| YDR074W | YDR080W | YDL074C | YDR009W | YDL002C | YDL006W | YFR025C | YGL154C |
| YDR080W | YDR127W | YDR009W | YDR034C | YDL006W | YDR009W | YGL024W | YGR183C |
| YDR162C | YDR162C | YDR049W | YDR127W | YDL074C | YDR034C | YGL154C | YHR116W |
| YDR207C | YDR207C | YDR162C | YDR159W | YDL074C | YDR127W | YGR183C | YIL020C |
| YDR290W | YDR290W | YDR290W | YDR234W | YDR009W | YDR159W | YHR116W | YJL071W |
| YDR316W | YDR316W | YDR375C | YDR290W | YDR080W | YDR162C | YIL020C | YJL088W |
| YDR375C | YDR388W | YDR388W | YDR388W | YDR127W | YDR234W | YJL071W | YJL204C |
| YDR388W | YEL013W | YDR500C | YDR532C | YDR162C | YDR290W | YJL088W | YJR090C |
| YEL013W | YEL061C | YDR532C | YEL013W | YDR269C | YDR316W | YJL204C | YLR038C |
| YER061C | YEL062W | YEL013W | YEL062W | YDR289C | YDR388W | YJR090C | YLR182W |
| YER092W | YER061C | YER061C | YER153C | YDR290W | YDR532C | YLR038C | YLR218C |
| YER151C | YER092W | YER083C | YHL007C | YDR316W | YEL013W | YLR218C | YLR337C |
| YER153C | YER153C | YER092W | YHR081W | YDR388W | YER151C | YLR239C | YMR282C |
| YFL036W | YFL036W | YER153C | YHR116W | YDR469W | YER153C | YLR337C | YNL315C |
| YGL024W | YFR025C | YFL007W | YJL088W | YDR532C | YFR025C | YLR420W | YNR050C |
| YGL043W | YGL024W | YFL036W | YJR090C | YEL013W | YGL024W | YLR442C | YOL009C |
| YGL045W | YGL026C | YGL045W | YKL184W | YER061C | YGL154C | YML106W | YOL108C |
| YGL046W | YGL043W | YGL219C | YKL212W | YER083C | YGR078C | YMR282C | YOL115W |
| YGL105W | YGL046W | YGL244W | YLR038C | YER092W | YGR105W | YNL315C | YOR065W |
| YGL136C | YGL066W | YGR183C | YLR061W | YER151C | YGR188C | YOL115W | YOR125C |
| YGL167C | YGL105W | YHR067W | YLR081W | YER153C | YHR116W | YOR065W | YPL172C |
| YGL168W | YGL212W | YHR116W | YLR218C | YFL013C | YIL090W | YOR125C | YPL180W |
| YGL212W | YGL219C | YIR023W | YLR337C | YFL036W | YJL071W | YOR221C | YPR191W |
| YGL219C | YGL255W | YJL147C | YLR338W | YFR025C | YJL088W | YPL172C |  |
| YGL244W | YGR188C | YJL204C | YML048W | YGL024W | YJL148W | YPL180W |  |
| YGR105W | YHL025W | YKL184W | YML063W | YGL043W | YJL204C | YPR134W |  |
| YGR183C | YHL025W | YKL212W | YMR269W | YGL045W | YJR090C | YPR191W |  |
| YGR188C | YHR021C | YLR015W | YMR282C | YGL046W | YKL212W | YDR227W |  |
| YGR229C | YHR041C | YLR038C | YNL021W | YGL149W | YLR038C |  |  |
| YHR067W | YHR081W | YLR056W | YNL120C | YGL154C | YLR081W |  |  |
| YHR116W | YHR116W | YLR081W | YNL147W | YGL167C | YLR182W |  |  |
| YHR189W | YHR199C | YLR192C | YNL265C | YGL168W | YLR218C |  |  |
| YIR023W | YJL029C | YLR218C | YNL315C | YGL212W | YLR337C |  |  |
| YJL029C | YJL088W | YLR239C | YNR010W | YGL219C | YLR338W |  |  |
| YJL046W | YKL184W | YLR337C | YOL001W | YGL244W | YLR357W |  |  |
| YJL101C | YKL212W | YLR338W | YOL009C | YGR078C | YLR420W |  |  |
| YJL204C | YKL216W | YML013C-A | YOL108C | YGR105W | YML048W |  |  |
| YKL184W | YLR038C | YML048W | YOL115W | YGR122W | YMR105C |  |  |
| YKL212W | YLR039C | YML063W | YOR065W | YHR030C | YMR165C |  |  |
| YLR038C | YLR061W | YML090W | YOR078W | YHR067W | YMR282C |  |  |
| YLR039C | YLR081W | YML121W | YOR125C | YHR116W | YNL021W |  |  |
| YLR056W | YLR182W | YMR063W | YOR141C | YJL071W | YNL120C |  |  |
| YLR061W | YLR218C | YMR269W | YPL172C | YJL101C | YNL265C |  |  |
| YLR081W | YLR318W | YMR282C | YPL180W | YJL147C | YOL001W |  |  |
| YLR182W | YLR337C | YMR312W | YPL213W | YJL154C | YOL009C  YOL108C |  |  |
| YLR192C | YLR338W | YNL021W | YPL226W | YJL204C | YOR065W |  |  |
| YLR218C | YLR357W | YNL119W | YPL248C | YKL184W | YOR070C |  |  |
| YLR239C | YLR393W | YNL120C | YPR024W | YLR015W | YOR125C |  |  |
| YLR262C | YLR420W | YNL169C | YPR173C | YLR038C | YOR334W |  |  |
| YLR337C | YML048W | YNL215W | YPR179C | YLR081W | YPL060W |  |  |
| YLR393W | YML097C | YNL236W | YPR191W | YLR182W | YPL172C |  |  |
| YML001W | YML106W | YNL265C |  | YLR192C | YPL178W |  |  |
| YML013C-A | YMR063W | YNL315C |  | YLR218C | YPL180W |  |  |
| YML048W | YMR105C | YNR006W |  | YLR239C | YPL213W |  |  |
| YML063W | YMR224C | YNR010W |  | YLR337C | YPL226W |  |  |
| YML097C | YMR269W | YOL001W  YOL115W |  | YML048W | YPL248C |  |  |
| YMR021C | YMR282C | YOL009C |  | YML090W | YPR134W |  |  |
| YMR063W | YNL064C | YOL108C |  | YMR021C | YPR173C |  |  |
| YMR105C | YNL081C | YOR065W |  | YMR063W | YPR179C |  |  |
| YMR269W | YNL236W | YOR070C |  | YMR105C |  |  |  |
| YMR282C | YNL315C | YOR125C |  | YMR154C |  |  |  |
| YNL021W | YNR010W | YOR141C |  | YMR165C |  |  |  |
| YNL081C | YNR050C | YOR196C |  | YMR282C |  |  |  |
| YNL119W | YOL004W | YOR221C |  | YMR312W |  |  |  |
| YNL215W | YOL009C | YOR275C |  | YNL021W |  |  |  |
| YNL236W | YOL058W | YPL086C |  | YNL052W |  |  |  |
| YNL315C | YOL081W | YPL172C |  | YNL080C |  |  |  |
| YNR006W | YOR026W | YPL178W |  | YNL119W |  |  |  |
| YNR010W | YOR030W | YPL226W |  | YNL120C |  |  |  |
| YOL009C | YOR065W | YPL248C |  | YNL215W |  |  |  |
| YOR026W | YOR070C | YPR134W |  | YNR006W |  |  |  |
| YOR030W | YOR078W | YPR173C |  | YNR010W |  |  |  |
| YOR065W | YOR080W | YPR179C |  | YOL001W |  |  |  |
| YOR068C | YOR106W | YPR191W |  | YOL009C |  |  |  |
| YOR069W | YOR125C |  |  | YOL012C |  |  |  |
| YOR070C | YOR132W |  |  | YOL108C |  |  |  |
| YOR125C | YOR196C |  |  | YOR030W |  |  |  |
| YOR132W | YOR235W |  |  | YOR065W |  |  |  |
| YOR196C | YOR275C |  |  | YOR068C |  |  |  |
| YOR235W | YOR293W |  |  | YOR070C |  |  |  |
| YOR275C | YOR309C |  |  | YOR070C |  |  |  |
| YOR293W | YPL172C |  |  | YOR125C |  |  |  |
| YPL172C | YPL180W |  |  | YOR196C |  |  |  |
| YPL226W | YPL213W |  |  | YOR221C |  |  |  |
| YPL248C | YPL226W |  |  | YOR275C |  |  |  |
| YPR008W | YPL239W |  |  | YOR334W |  |  |  |
| YPR024W | YPL248C |  |  | YPL055C |  |  |  |
| YPR070W | YPR008W |  |  | YPL060W |  |  |  |
| YPR134W | YPR024W |  |  | YPL086C |  |  |  |
| YPR173C | YPR134W |  |  | YPL172C |  |  |  |
| YPR179C | YPR173C |  |  | YPL226W |  |  |  |
| YPR191W | YPR179C |  |  | YPL248C |  |  |  |
|  | YPR191W |  |  | YPR070W |  |  |  |
|  |  |  |  | YPR134W |  |  |  |
|  |  |  |  | YPR173C |  |  |  |
|  |  |  |  | YPR179C |  |  |  |

| Table S4. Analysed SDL Interactions from two replicates of the tag only screen | |
| --- | --- |
| Tag only Rep.1 | **Tag only Rep.2** |
| YBR018C | YBR018C |
| YBR019C | YBR019C |
| YBR020W | YBR020W |
| YBR020W | YBR020W |
| YCR028C-A | YCR028C-A |
| YCR071C | YCR071C |
| YDL104C | YDL104C |
| YER083C | YER153C |
| YER153C | YHL003C |
| YGL024W | YHR116W |
| YHR116W | YIL094C |
| YLR038C | YKL139W |
| YLR114C | YLR114C |
| YLR393W | YMR135W-A |
| YML090W | YMR282C |
| YMR021C | YNL120C |
| YMR282C | YNL218W |
| YNL052W | YNL315C |
| YNL315C | YOR033C |
| YOL004W | YOR202W |
| YOL008W | YOR202W |
| YOR033C | YOR358W |
| YOR037W | YPL172C |
| YOR202W | YPL248C |
| YOR358W |  |
| YPL172C |  |
| YPL248C |  |
| YPL262W |  |
| YPR191W |  |
